# γ-Aminobutyric acid production by selected lactic acid bacteria isolate of an Indonesian indigenous fermented buffalo milk (*dadih*) origin

**DOI:** 10.14202/vetworld.2019.1352-1357

**Published:** 2019-08-30

**Authors:** Harnentis Harnentis, Nurmiati Nurmiati, Yetti Marlida, Frederick Adzitey, Nurul Huda

**Affiliations:** 1 Department of Animal Nutrition and Feed Technology, Faculty of Animal Science, Andalas University, West Sumatera, Indonesia; 2 Department of Biology, Faculty of Natural Sciences, Andalas University, West Sumatera, Indonesia; 3 Department of Veterinary Science, Faculty of Agriculture, University for Development Studies, Box TL 1882, Tamale, Ghana; 4 Department of Food Science, Faculty of Food Science and Nutrition, Universiti Malaysia Sabah, Kota Kinabalu, Sabah, Malaysia

**Keywords:** fermented buffalo milk, Indonesian indigenous product, lactic acid bacteria, γ-aminobutyric acid

## Abstract

**Aim:**

This study aimed at optimizing γ-aminobutyric acid (GABA) production using lactic acid bacteria (LAB) of an Indonesian indigenous fermented buffalo milk (*dadih*) origin. This study utilized LAB previously cultured from *dadih* that has the ability to produce GABA.

**Materials and Methods:**

The study started with the identification of selected LAB by 16S rRNA, followed by optimization of GABA production by culture conditions using different initial pH, temperature, glutamate concentration, incubation time, carbon, and nitrogen sources. 16S rRNA polymerase chain reaction and analysis by phylogenetic were used to identify *Lactobacillus plantarum* (coded as N5) responsible for the production of GABA.

**Results:**

GABA production by high-performance liquid chromatography was highest at pH of 5.5, temperature of 36°C, glutamate concentration of 500 mM, and incubation time of 84 h. Peptone and glucose served as the nitrogen and carbon sources, respectively, whereas GABA was produced at optimum fermentation condition of 211.169 mM.

**Conclusion:**

Production of GABA by *L. plantarum* N5 was influenced by initial pH of 5.5, glutamic acid concentration, nitrogen source, glucose as carbon source, and incubation temperature and time.

## Introduction

Indigenous fermented buffalo milk, locally known as *dadih*, is an essential food source for the populace of West Sumatera, Jambi, and Riau of Indonesia. Putra *et al*. [1] indicated that *dadih* is an important diet and consumed largely by people of West Sumatera and Minangkabau. Microflora of *dadih* are essential for their role in fermentation (aroma, texture, and acidity), therapeutic (improves digestion), and antimicrobial activity [2]. *Dadih* generally can be consumed directly or with rice. At first glance, this food seems unfamiliar to some Indonesian people. *Dadih* itself comes from buffalo milk which is kept in bamboo and covered using banana leaves. It is then allowed to stand at room temperature for a day to form clots. According to Surono [3], clumping occurs due to the presence of microbes derived from bamboo and banana leaves so that it will produce a form that is clad and yellowish-white and has a distinctive aroma. Pato [4] stated that *dadih* contains high protein (39.8%) with complete essential amino acids, calcium, Vitamin B, and Vitamin K which are formed during the fermentation process. In addition, bacteria in *dadih* are capable of inhibiting intestinal pathogens and thus can help facilitate digestion. Nowadays, interest in indigenous fermented buffalo milk and microflora is increasing, especially in the production of metabolites that can be used as food or feed additive, such as γ-aminobutyric acid (GABA).

Lactic acid bacteria (LAB) such as *Lactobacillus plantarum, Lactobacillus brevis*, *Streptococcus agalactiae, Bacillus cereus, Streptococcus uberis* [5], and *L. plantarum* [6] have been isolated from *dadih*. Surono [3] also reported the isolation of LAB such as *Lactococcus lactis*, *L. brevis*, *Lactococcus casei, L. plantarum, E. faecium*, and *Leuconostoc* *mesenteroides* from *dadih*. Functional compounds such as peptides and oligosaccharides together with lactic acid are produced when LAB are used to ferment foods. In addition, functional compounds like GABA (a non-protein amino acid) functions as neurotransmitter inhibitor and exhibit hypotensivity [7]. GABA is one of the most important functional components in fermented foods due to its physiological functions such as neurotransmission and antihypertensive activities [8], and anti-heat stress for broilers [9]. Nonetheless not much is known about GABA-rich fermented foods that can be used as feed additives. GABA production by microbes is affected by factors such as initial pH, fermentation time, medium composition, glutamate concentration, and temperature [7,8]. Li *et al*. [10] added that the highest GABA production by *L. brevis* was achieved at optimum pH of 5.0. Komatsuzaki *et al*. [11] found the optimum GABA production at 500 mM glutamate content in culture media of *Lactobacillus paracasei* NFRI 7415. Yang *et al*. [12] reported that optimizing fermentation conditions to a pH of 4.5 resulted in an improved GABA production in *Streptococcus salivarius* culture.

This study was conducted to improve the production of GABA by selected LAB that were isolated from indigenous fermented buffalo milk (*dadih*) through optimization of fermentation parameters such as initial pH, temperature, glutamate concentration, incubation time, carbon, and nitrogen sources.

## Materials and Methods

### Ethical approval

No human or animal objects were used; therefore ethical approval was not sought.

### Isolation of LAB

The LAB strains used were obtained from indigenous fermented buffalo milk (*dadih*) in West Sumatera region, Indonesia [13].

### Identification of LAB by 16S rRNA

Identification of LAB by 16S rRNA was done using 63 F: 5’-CAG GCC TAA CAC ATG CAA GTC-3’ and 1387 R: 5’-GGG CGG GGT GTA CAA GGC-3’. An approximately 1.5 kb fragment was amplified in a Biometra’s T-Personal Thermal Cycler, USA. The polymerase chain reaction (PCR) conditions were as follows: Initial denaturation at 95°C for 5 min, followed by 35 cycles of denaturation at 94°C for 1 min, annealing at 56°C for 1 min, and a final extension at 72°C for 1.5 min. The PCR products were analyzed on 1.0% (w/v) agarose gel electrophoresis (Mupid-Exu Submarine Electrophoresis System, Advance) in 1× tris-acetate-EDTA buffer at 100 V for 30 min. It was visualized on a gel documentation system (Biodoc Analyze, Biometra, USA). Purified PCR products were sequenced with 16S rRNA primers. Sequences of the whole gene fragment were used for similarity search against NCBI GenBank database using the Basic Local Alignment Search Tool (BLAST) program available at website http://blast.ncbi.nlm.nih.gov/Blast.cgi.

### Selection of LAB-producing GABA

The previous study by Marlida *et al*. [13] found a sum of 10 LAB of *dadih* origin which exhibited a potent capacity to produce GABA-based on thin-layer chromatography (TLC) and spectrophotometer. The 10 LAB were cultivated in 10 ml of MRS Broth (Merck) having a glutamic acid concentration of 50 mM, a pH of 5 and incubated at 30°C for 72 h. GABA content of the culture in the MRS Broth (1 ml) was measured and used for high-performance liquid chromatography (HPLC) analysis.

### Optimization GABA production

The optimization of GABA production was done to determine the influence of fermentation conditions such as pH (3.5-7), glutamic acid concentration (0-600 mM), culture temperature (30-42°C), incubation time (0-108 h), 0.1-0.9% inoculum level (10^9^ CFU/ml), 3% carbon (w/v), and 0.3% nitrogen (w/v) sources on GABA production by selected LAB. HPLC was used to measure the GABA content in the supernatants. Chemicals involved in this optimization are glutamic acid (Sigma-Aldrich, 99%), glucose (Merck, pure), maltose (Merck, pure), sucrose (Merck, pure), NH_4_NO_3_ (Merck, pure), peptone (Merck, pure), skim milk (Intrasol), and yeast extract (Merck, pure).

### Identification and quantification of GABA by HPLC

GABA produced after fermentation was measured using HPLC (Agilent Tech, Waldron, Germany). The HPLC had Hypersil ODS C18 reverse-phase column with 250 mm length, 5 μm diameter, and 4.6 mm width. The culture broth (100 μL) was sieved using a 0.22 μm filter. Mobile phase A of the HPLC was filled with 10.254 g of 99% sodium acetate three hydrates (Sigma-Aldrich, USA). It was then dissolved in 900 mL of deionized water and 500 μL trimethylamine (Merck). Glacial acetic was used to adjust the pH of the mobile phase A to 5.8. Acetonitrile (HPLC grade, Merck) and deionized water were the mobile phases B and C, respectively. Phases were filtered through 0.22 μm membrane filter, with an injection volume of 20 μL. The final compound was identified by the detector at 254 nm. The quantity of GABA produced was computed by comparing the peak to the GABA standard.

## Results

### Identification of selected LAB-producing GABA

**Figure-1:**
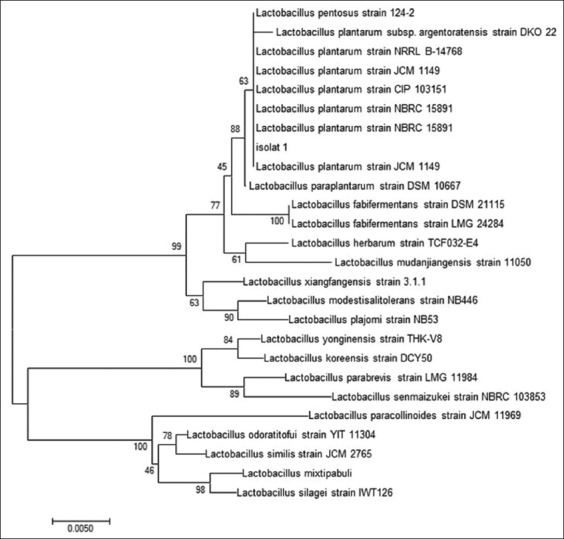
Phylogenetic tree of 16S rRNA gene of *Lactobacillus plantarum* N5 isolated from fermented buffalo milk (*dadih*) using neighbor-joining method MEGA 7.0.

After qualitative and quantitative screening using TLC and HPLC, respectively [13], one isolate (isolate N5) showed the highest GABA production of 47.2 mM, compared to N1: 30.915; N2: 31.515; N3: 9.915; N4: 14.315; N6: 18.015; N7: 31.215; N8: 17.665; N9: 25.815, and N10: 7.815 mM. Isolate N5 was identified by PCR of 16S rRNA gene sequences and a phylogenetic analysis was constructed for this isolate to compare it to homologous strains. N5 originated from fermented buffalo milk (*dadih*), had a sequence length of 1400 bp, and was identified as *L. plantarum*. Analysis of *L. plantarum* N5 using phylogenetic tree revealed similarities with *L. plantarum* strains NBRC 15891 (99%) and JCM1149 (99%). BLAST was then used to align the sequences to obtain LAB isolates of similar sequences to *L. plantarum* N5. Following kinship analysis, MEGA 7.0 (Society for Molecular Biology and Evolution, USA) was used to draw a phylogenetic tree for *L. plantarum* N5 and 25 homology LAB, as shown in Figure-1.

### Effect of temperature and initial pH

**Figure-2:**
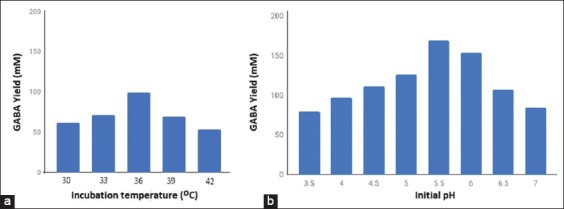
Effect of incubation temperature (a) and initial pH of medium (b) on γ-aminobutyric acid production by *Lactobacillus plantarum* N5.

Figure-2a reveals the effect of temperature (30-42°C) on GABA yield. This was obtained using glutamic acid concentration of 50 mM, initial pH of 5, and incubation time of 72 h. Figure-2a shows that the optimum GABA production was at a temperature of 36°C and yielded 99.218 mM of GABA. GABA yield decreased with increased temperature (beyond 36°C). Figure-2b reveals the effect of initial pH on GABA yield. The initial pH profile was linear with GABA production from pH 3.5 to 5.5, whereas the optimum production of GABA was at pH 5.5. Increased of pH from 6.0 to 7.0 decreased GABA production by *L. plantarum* N5. pH mainly regulates the biosynthesis of GABA and this process is species-dependent because LAB GAB enzyme has diverse characteristics.

### Effect of incubation time and L-glutamate concentration

**Figure-3:**
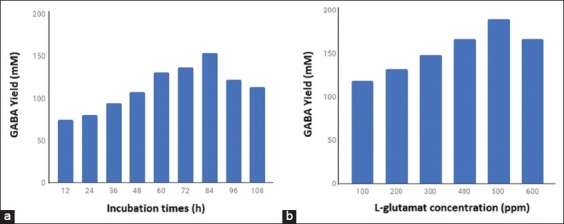
Effect of incubation time (a) and L-glutamate concentration (b) on γ-aminobutyric acid production by *Lactobacillus plantarum* N5.

The incubation time and L-glutamate concentration on GABA production were done using an initial pH of 5.5, culture temperature of 36°C, and incubation time of 72 h in the culture medium. Figure-3a presents the effect of incubation time on the production of GABA, which shows that the optimum production of GABA was obtained at 84 h, if incubation time is increased to 108 h a decrease in GABA occurs. Figure-3b shows that the optimum glutamic acid for highest GABA production was 500 mM, whereas increasing glutamic acid to 600 mM decreased GABA production.

### Effect of carbon and nitrogen sources

**Figure-4:**
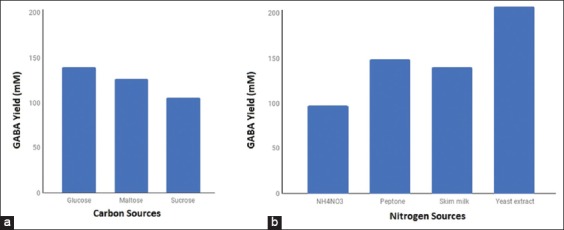
Effect of carbon (a) and nitrogen (b) sources on the γ-aminobutyric acid production by *Lactobacillus plantarum* N5.

Carbon and nitrogen are important compounds required for the growth of *L. plantarum* N5 for optimum GABA production. Figures-4a and b show that glucose and yeast extract are good sources of carbon and nitrogen, respectively, for high production of GABA.

## Discussion

### Identification of selected isolate

This study revealed that *L. plantarum* N5 was genetically closer (similarity of 99%) to *L. plantarum* strain NBRC 15891 and *L. plantarum* strain JCM1149. In determining the genetic differences and variations in populations, genetic distances can be used [14]. This is calculated based on DNA sequences from the number of differences in the polymorphic gene loci of each population. Furthermore, phylogenetic analysis is important in sequence analysis [15]. Phylogenetics also provide relevant information required to understand changes that occur during evolution of different organisms.

### Effect of temperature and initial pH

This is due to the fact that increasing temperature affect the growth rate of *L. plantarum* N5. Besides that, the enzyme produced in the medium such as glutamic acid decarboxylase (GAD) enzyme can influence the growth of *L. plantarum* N5. The function of the GAD enzyme is to convert glutamic acid to GABA, when the GAD activity is low, the resulting GABA will be low (52.997 mM at 42°C). Effects of temperature on the production of GABA have been reported by other researchers. The production of GABA by *L. brevis* RK03 was highest at a temperature of 30°C and yielded, 21.936 mg/L [16]. *L. brevis* NM101-1 and *L. plantarum* DSM749 had optimal temperature of 35°C for highest GABA yield of 168.58 mM and 140.69 mM, respectively [17]. *L. plantarum* Taj-Apis362 produced the highest GABA at a temperature of 36°C [18]. Thermodynamic equilibrium of a reaction is affected by many factors including temperature. The right culture temperature and cell density are required for efficient conversion of glutamate to GABA. Besides, the incubation temperature, biocatalyst activity, and stability are essential factors that affect maximum GABA yield during fermentation.

Initial pH of the medium for fermentation is an important condition in the production of GABA and has a relationship with GAD activity. Optimum pH is required for maintaining the activities of GAD, an enzyme responsible for GABA synthesis [11]. Low or high pH may lead to partial loss of GAD activities. In this study, the highest GABA production was obtained at a pH of 5.5. Yip *et al*. [19] and Fatemi *et al*. [20] showed that enhancing GABA production in an acidic condition is closely linked to the characteristics of GAD which shows enhanced activity and stability when hydrogen ions are present.

### Effect of incubation time and L-glutamate concentration

Figure-3a shows that GABA production increased rapidly during 60-84 h of incubation, optimum at 84 h, and decreased after 84 h. Biosynthesis of GABA production might be attributed to inhibitory effects of glutamic acid and the concentration of GABA [21-23]. In Figure-3a, it can also be seen that the transformation of glutamic acid to GABA by GAD follows the growth pattern of *L. plantarum* N5, at a fermentation time of 12-24 h for the lag phase, 36-84 h for the exponential phase, and 96-108 h for the stationary phase. In the stationary phase, lower GABA production caused decreased in the nutrients needed for growth and enzyme production. Figure-3b shows a higher GABA yield at 500 mM of glutamic acid when the concentration was increased to 600 mM, the production of GABA decreased. Li *et al*. [10] also found that the production of GABA was suppressed when glutamic acid concentration was increased.

### Effect of carbon and nitrogen sources

Figures-4a and b show the effects of carbon and nitrogen sources on the production of GABA by selected LAB. In Figure-4a, MRS Broth was used to investigate the effects of carbon sources on the production of GABA by *L. plantarum*. The MRS Broth contained 3% carbon (w/v) and 500 mM L-glutamic acid. There were remarkable differences in GABA production due to the addition of carbon of different sources. Glucose was found to be the best carbon source for the production of GABA (139.843 mM) followed by maltose (126.649 mM) and sucrose (106.033 mM). Similarly to this study, glucose was found to be the best carbon source for the production of GABA [22,24].

With regard to sources of nitrogen, yeast extract was found to be the best for the production of GABA (211.69 mM), followed by peptone (151.698 mM), skim milk (142.636 mM), and NH_4_NO_3_ (99.216 mM), which might be attributed to their compositions in the MRS Broth. Researchers have shown that diverse carbon sources can be optimized for GABA production such as 3% sucrose for *L. brevis* 340G, 4% sucrose for *Lactobacillus sakei* B2-16, and 1% glucose for *L. buchneri* MS[22,25,26].

## Conclusion

The conditions optimum for maximum production of GABA by *L. plantarum* N5 were an initial pH of 5.5, glutamic acid concentration of 500 mM, yeast extract as nitrogen source, glucose as carbon source, and incubation temperature and time of 36°C and 84 h, respectively.

## Authors’ Contributions

HH, NN, and YM collected data and wrote the manuscript. YM designed the study. FA and NH reviewed and updated the manuscript. All authors read and approved the final manuscript.
